# Revised STandards for Reporting Interventions in Clinical Trials of Acupuncture (STRICTA): Extending the CONSORT Statement

**DOI:** 10.1371/journal.pmed.1000261

**Published:** 2010-06-08

**Authors:** Hugh MacPherson, Douglas G. Altman, Richard Hammerschlag, Li Youping, Wu Taixiang, Adrian White, David Moher, on behalf of the STRICTA Revision Group

**Affiliations:** 1 Department of Health Sciences, University of York, York, United Kingdom; 2 Centre for Statistics in Medicine, University of Oxford, Oxford, United Kingdom; 3 Department of Research, Oregon College of Oriental Medicine, Portland, Oregon, United States of America; 4 Chinese Cochrane Centre, Chinese Evidence-Based Medicine Centre, West China Hospital, Sichuan University, China; 5 Primary Care Research, Peninsula Medical School, Universities of Exeter and Plymouth, Plymouth, United Kingdom; 6 Ottawa Methods Centre, Ottawa Hospital Research Institute; Department of Epidemiology and Community Medicine, Faculty of Medicine, University of Ottawa, Ottawa, Canada

## Abstract

Hugh MacPherson and colleagues present an updated reporting guideline called STRICTA, which stands for Revised STandards for Reporting Interventions in Clinical Trials of Acupuncture.

**Summary Points**

- The Standards for Reporting Interventions in Clinical Trials of Acupuncture (STRICTA) were published in five journals in 2001 and 2002. These guidelines, in the form of a checklist and explanations for use by authors and journal editors, were designed to improve reporting of acupuncture trials, particularly the interventions, thereby facilitating their interpretation and replication. Subsequent reviews of the application and impact of STRICTA have highlighted the value of STRICTA as well as scope for improvements and revision.
- To manage the revision process a collaboration between the STRICTA Group, the CONSORT Group, and the Chinese Cochrane Centre was developed in 2008. An expert panel with 47 participants was convened that provided electronic feedback on a revised draft of the checklist. At a subsequent face-to-face meeting in Freiburg, a group of 21 participants further revised the STRICTA checklist and planned dissemination.
- The new STRICTA checklist, which is an official extension of CONSORT, includes six items and 17 sub-items. These set out reporting guidelines for the acupuncture rationale, the details of needling, the treatment regimen, other components of treatment, the practitioner background, and the control or comparator interventions. In addition, and as part of this revision process, the explanations for each item have been elaborated, and examples of good reporting for each item are provided. In addition, the word “controlled” in STRICTA is replaced by “clinical”, to indicate that STRICTA is applicable to a broad range of clinical evaluation designs, including uncontrolled outcome studies and case reports.
- It is intended that the revised STRICTA, in conjunction with both the main CONSORT Statement and extension for nonpharmacologic treatment, will raise the quality of reporting of clinical trials of acupuncture.

## Introduction

The STRICTA (Standards for Reporting Interventions in Clinical Trials of Acupuncture) reporting guidelines, first published in 2001 [1]–[9], were designed to improve the completeness and transparency of reporting of interventions in controlled trials of acupuncture, in order that such trials may be more accurately interpreted and readily replicated. STRICTA comprised a checklist that expanded the generic content of Item 4 of the CONSORT statement [10],[11], which relates to the reporting of the intervention.

A survey of authors of clinical trials and systematic reviews was subsequently conducted to determine the usefulness of STRICTA in helping them write their reports [12]. In addition, a survey of 90 acupuncture trials was undertaken to assess whether use of the STRICTA checklist was associated with improved reporting over time [13]. The results of these initiatives led to conclusions that most STRICTA items were found to be necessary and easy to use, though some were seen as poorly reported, ambiguous or possibly redundant, and a number of suggestions were made for additional items. A revision of STRICTA was therefore proposed.

Meanwhile, extensions to CONSORT have been developed to cover the reporting of non-pharmacological treatments [14],[15] and pragmatic trials [16]. Since there are acupuncture specific aspects to reporting not covered by these extensions, it was decided that STRICTA should be revised in a manner congruent with CONSORT and its extensions for non-pharmacological treatments and pragmatic trials.

The combination of these developments led to an agreement between the CONSORT Group and the STRICTA Group, in collaboration with the Chinese Cochrane Centre and the Chinese Centre for Evidence-based Medicine, to revise STRICTA as a formal extension to CONSORT. The revision processes have been described in more detail elsewhere [17]. This paper describes the outcome in terms of a new checklist, updated explanations, and published examples of good reporting.

## Methods

In the summer of 2008, a group of 47 experts from the original STRICTA Group, the CONSORT Group, the World Federation of Acupuncture and Moxibustion Societies, the Acupuncture Trialists' Collaboration [18], the Society for Acupuncture Research [19], and clinical trial authors were surveyed [12]. The experts were from 15 countries, 41 had academic positions, 31 were acupuncturists, 18 were involved with journals, such as board members, 15 were physicians, and 11 had been involved previously in developing reporting guidelines. These experts were consulted in regard to a draft of revised STRICTA items that had evolved from previous research [12],[13]. Feedback was collated and forwarded (with permission) to those invited to a consensus development workshop, the next phase of the revision process.

Twenty-one individuals attended a workshop in Freiburg, Germany, in October 2008. The attendees included experts in epidemiology, trial methodology, statistics, and medical journal editing. Just over half the participants were acupuncturists from a variety of backgrounds, including physician and non-physician. All attendees received collated feedback from the 47 experts, along with a draft revised STRICTA checklist for consideration.

The workshop comprised presentations about the history of STRICTA, CONSORT, and the then new CONSORT non-pharmacological treatments extension [14],[15]. The results of two investigations into the utility and acceptability of STRICTA [12],[13], and the subsequent consultation with the 47 experts, were also presented. A general discussion and agreement on generic issues relating to STRICTA were followed by a discussion of each nominated checklist item. The aim was to agree, where possible, on the content of the updated draft checklist as well as to develop a revised set of explanations for each included item.

Subsequent to the workshop, a small writing group edited drafts of the revised STRICTA checklist, identifying for each item one or more exemplars of good reporting, and developed text explaining the rationale and discussing relevant evidence. Taking into account further feedback from those attending the Freiburg workshop, the writing group finalised the STRICTA checklist, the explanations and the examples of good reporting.

## Results

*Note: In the Examples that follow, the embedded terms (ref) and (refs) refer to sources that are reported in the original published studies, but the details of these sources are not provided in this article for reasons of brevity.*

There was agreement that STRICTA should continue to function as a stand-alone guideline for reporting acupuncture studies, and be an official extension of CONSORT for reporting randomized controlled trials. There was also consensus on a minor change of name, in that the word “controlled” in STRICTA should be replaced by “clinical”, to indicate that it was applicable for reporting a broad range of clinical evaluation designs, including uncontrolled outcome studies and case reports. The group agreed that the rationale behind reporting should be to provide the information needed to allow replication of a study, reduce ambiguity and enhance transparency. The group recognised that acupuncture trials inevitably differ in the degree of individualisation of care that is permitted, and agreed that the reporting guideline should acknowledge this and be applicable across the whole range of designs. The group also suggested that the revised STRICTA statement, when published, should be presented as embedded within the two-group parallel trial CONSORT checklist [10] and its non-pharmacological treatment extension checklist [14].

The revised STRICTA checklist comprises six items broken out into seventeen sub-items (Table 1). Table 2 presents how the revised STRICTA checklist fits within the CONSORT checklist [10] and its extension for non-pharmacological treatments [14]. Below we provide the checklist text for each of the six items and their sub-items, as well as explanations on the need for their adequate reporting and examples of good reporting from the published literature.

**Table 1:** STRICTA 2010 checklist of information to include when reporting interventions in a clinical trial of acupuncture.

Item	Detail
**1. Acupuncture rationale**	1a) Style of acupuncture (e.g. Traditional Chinese Medicine, Japanese, Korean, Western medical, Five Element, ear acupuncture, etc)
	1b) Reasoning for treatment provided, based on historical context, literature sources, and/or consensus methods, with references where appropriate
	1c) Extent to which treatment was varied
**2. Details of needling**	2a) Number of needle insertions per subject per session (mean and range where relevant)
	2b) Names (or location if no standard name) of points used (uni/bilateral)
	2c) Depth of insertion, based on a specified unit of measurement, or on a particular tissue level
	2d) Response sought (e.g. *de qi* or muscle twitch response)
	2e) Needle stimulation (e.g. manual, electrical)
	2f) Needle retention time
	2g) Needle type (diameter, length, and manufacturer or material)
**3. Treatment regimen**	3a) Number of treatment sessions
	3b) Frequency and duration of treatment sessions
**4. Other components of treatment**	4a) Details of other interventions administered to the acupuncture group (e.g. moxibustion, cupping, herbs, exercises, lifestyle advice)
	4b) Setting and context of treatment, including instructions to practitioners, and information and explanations to patients
**5. Practitioner background**	5) Description of participating acupuncturists (qualification or professional affiliation, years in acupuncture practice, other relevant experience)
**6. Control or comparator interventions**	6a) Rationale for the control or comparator in the context of the research question, with sources that justify this choice
	6b) Precise description of the control or comparator. If sham acupuncture or any other type of acupuncture-like control is used, provide details as for Items 1 to 3 above.

Note: This checklist, which should be read in conjunction with the explanations of the STRICTA items provided in the main text, is designed to replace CONSORT 2010's item 5 when reporting an acupuncture trial.

**Table 2:** CONSORT 2010 checklist with the Non-pharmacological Trials Extension to CONSORT (with STRICTA 2010 extending CONSORT Item 5 for acupuncture trials).

Section/Topic	Item #	CONSORT 2010 Statement*: Checklist item [10]. Describe:	Additional items from the Non-pharmacological Trials Extension to CONSORT [14]. Add:
*TITLE AND ABSTRACT*			
	1.a	Identification as a randomized trial in the title	In the abstract, description of the experimental treatment, comparator, care providers, centres and blinding status.
	1.b	Structured summary of trial design, methods, results, and conclusions; for specific guidance see CONSORT for Abstracts [58],[59]	
*INTRODUCTION*			
Background and objectives	2.a	Scientific background and explanation of rationale	
	2.b	Specific objectives or hypotheses	
*METHODS*			
Trial design	3.a	Description of trial design (e.g., parallel, factorial) including allocation ratio	
	3.b	Important changes to methods after trial commencement (e.g. eligibility criteria), with reasons	
Participants	4.a	Eligibility criteria for participants	When applicable, eligibility criteria for centers and those performing the interventions.
	4.b	Settings and locations where the data were collected	
**Interventions**	**5**	The interventions for each group with sufficient details to allow replication, including how and when they were actually administered	Precise details of both the experimental treatment and comparator - see Table 1 for details
Outcomes	6.a	Completely defined pre-specified primary and secondary outcome measures, including how and when they were assessed	
	6.b	Any changes to trial outcomes after the trial commenced with reasons	
Sample size	7.a	How sample size was determined	When applicable, details of whether and how the clustering by care providers or centers was addressed.
	7.b	When applicable, explanation of any interim analyses and stopping guidelines	
Randomization			
*Sequence generation*	8.a	Method used to generate the random allocation sequence	When applicable, how care providers were allocated to each trial group.
	8.b	Type of randomization; details of any restriction (e.g., blocking and block size)	
*Allocation concealment*	9	Mechanism used to implement the random allocation sequence (e.g., sequentially numbered containers), describing any steps taken to conceal the sequence until interventions were assigned	
*Implementation*	10	Who generated the random allocation sequence, who enrolled participants, and who assigned participants to interventions	
Blinding	11.a	If done, who was blinded after assignment to interventions (e.g. participants, care providers, those assessing outcomes) and how	Whether or not those administering co-interventions were blinded to group assignment. If blinded, method of blinding and description of the similarity of interventions.
	11.b	If relevant, description of the similarity of interventions	
Statistical methods	12.a	Statistical methods used to compare groups for primary and secondary outcomes	When applicable, details of whether and how the clustering by care providers or centers was addressed.
	12.b	Methods for additional analyses, such as subgroup analyses and adjusted analyses	
*RESULTS*			
Participant flow (a diagram is strongly recommended)	13.a	For each group, the numbers of participants who were randomly assigned, received intended treatment, and were analyzed for the primary outcome	The number of care providers or centers performing the intervention in each group and the number of patients treated by each care provider or in each center.
	13.b	For each group, losses and exclusions after randomization, together with reasons	
Implementation of intervention			Details of the experimental treatment and comparator as they were implemented.
Recruitment	14.a	Dates defining the periods of recruitment and follow-up	
	14.b	Why the trial ended or was stopped	
Baseline data	15	A table showing baseline demographic and clinical characteristics for each group	When applicable, a description of care providers (case volume, qualification, expertise, etc.) and centers (volume) in each group.
Numbers analyzed	16	For each group, number of participants (denominator) included in each analysis and whether the analysis was by original assigned groups	
Outcomes and estimation	17.a	For each primary and secondary outcome, results for each group, and the estimated effect size and its precision (e.g., 95% confidence interval)	
	17.b	For binary outcomes, presentation of both absolute and relative effect sizes is recommended	
Ancillary analyses	18	Results of any other analyses performed, including subgroup analyses and adjusted analyses, distinguishing pre-specified from exploratory	
Harms	19	All important harms or unintended effects in each group; for specific guidance see CONSORT for Harms [60]	
*DISCUSSION*			
Limitations	20	Trial limitations, addressing sources of potential bias, imprecision, and, if relevant, multiplicity of analyses	
Generalizability	21	Generalizability (external validity, applicability) of the trial findings	Generalizability (external validity) of the trial findings according to the intervention, comparators, patients and care providers and centers involved in the trial.
Interpretation	22	Interpretation consistent with results, balancing benefits and harms, and considering other relevant evidence	In addition, take into account the choice of the comparator, lack of or partial blinding, unequal expertise of care providers or centers in each group.
*OTHER INFORMATION*			
Registration	23	Registration number and name of trial registry	
Protocol	24	Where the full trial protocol can be accessed, if available	
Funding	25	Sources of funding and other support (e.g., supply of drugs); role of funders	

*We strongly recommend reading this Statement in conjunction with the CONSORT 2010 explanation and elaboration [11] for important clarifications on all the items. If relevant, we also recommend reading CONSORT extensions for cluster randomized trials [61], noninferiority and equivalence trials [62], herbal interventions [63], and pragmatic trials [16]. Moreover, additional extensions are forthcoming. For those and also for up-to-date references relevant to this checklist, see http://www.consort-statement.org.

## Stricta Item 1: Acupuncture Rationale

### Item 1a

Style of acupuncture (e.g. Traditional Chinese Medicine, Japanese, Korean, Western medical, Five Element, ear acupuncture, etc.).

#### Explanation

Acupuncture has a long history in many cultures and is characterised by a broad diversity of styles and approaches in both East Asia and the West [20]. In order for the readers to contextualize the trial within the range of current clinical practices, researchers should state the overall style or approach on which they have based the treatments. If the researcher believes the treatment approach is completely novel, then this should be clearly stated.

#### Examples

(i) We based the acupuncture point selections on Traditional Chinese Medicine meridian theory to treat knee joint pain, known as the “Bi” syndrome [21].

(ii) Participants were randomised to two styles of acupuncture: Japanese style (Kiiko-Matsumoto's Form) and Traditional Chinese Medicine style [22].

(iii) Four out of five of the acupuncturists primarily practised the Five Element style with a diagnostic focus on individual ‘Causative Factors’,(ref) and one used the Traditional Chinese Medicine (TCM) style with diagnosis primarily based on syndrome patterns.(ref) Both styles are rooted in traditional acupuncture theory, and they are the most common traditional approaches used by professional acupuncturists in the UK today(ref) [23].

(iv) Each patient was treated with non-local needle acupuncture (according to the theory of channels of Traditional Chinese Medicine) at distant points, and dry needling of local myofascial trigger points [24].

### Item 1b

Reasoning for treatment provided, based on historical context, literature sources, and/or consensus methods, with references where appropriate.

#### Explanation

The author(s) should provide the reasoning for the chosen treatment, including rationale for diagnosis, point selection and treatment procedures. The “rules” that were used in providing treatments should be described. When treatments were selected that have roots in traditional practice, it is recommended that the historical and cultural context be supplied. This is relevant for interventions within styles such as “Traditional Chinese Medicine” or “TCM”, where the broad diversity of approaches requires careful identification of where and when the treatment parameters were developed. Where consensus methods, expert clinical panels, practitioner surveys or some combination of sources have been used to define the treatment protocol, it is recommended that full details of the methodology be given. Literature and other sources should be provided where relevant, in order that others can replicate the trial by consulting these source(s) and/or developmental methods on which treatment was based. Authors are encouraged to reference published works that are easily obtainable, such as a book or journal article. If the reference is a thesis, non-published work, written material only available in a different language from the journal article, or a verbal communication, authors are encouraged to present or summarise the information in an appendix or make it otherwise generally available (e.g. on a website). For fully individualised trials where the goal is to have representative practitioners who are encouraged to practice as they normally do, it is appropriate to specify the selection process for the practitioners, providing details of criteria for their inclusion. It is important to note that where details of the intended intervention are defined in advance, it is possible that what was actually administered may have differed. In such cases, precise details of the treatments that were provided are also necessary.

#### Examples

(i) This study employed a style of Japanese acupuncture developed by Shima and Chace (ref) and Manaka (ref), and follows the Japanese acupuncture training curriculum at the New England School of Acupuncture. In comparison to typical traditional Chinese medicine (TCM) acupuncture, Japanese acupuncture uses smaller needles and inserts needles less deeply and with less manipulation.(ref) For these reasons, we believed Japanese acupuncture would be less invasive than TCM, and thus better received by our adolescent population. Japanese acupuncture has been shown to be effective in treating certain pain conditions.(ref) The specific acupuncture protocols employed in this study are briefly described below and discussed in greater detail in a companion paper(ref) [25].

(ii) We based point selection on individualized Western acupuncture techniques by using a list of points previously reported as being effective in neck pain (refs) and by reaching a consensus according to our own clinical and teaching practice.(ref) The specific points for each individual were defined at each treatment session, depending on the patient's pain distribution and palpation of the neck and thorax to determine *ah-shi* points, or local tender points, for acupuncture. At least one distal point was used. Point location and depth of insertion were as described in traditional texts(ref) [26].

(iii) We developed the treatment strategies for acupuncture and minimal acupuncture in a consensus process with three acupuncture specialists (names provided) representing two major German societies for medical acupuncture: the German Medical Acupuncture Association (Deutsche Ärztegesellschaft für Akupunktur, DÄGfA) and the International Society for Chinese Medicine (Societas Medicinae Sinensis, SMS). The first step involved three specialists (names provided) and the study team developing a proposal, which was followed by a discussion including more than 30 acupuncture experts from both acupuncture societies. The final intervention strategies were defined by the above mentioned three specialists together with the study team and subsequently were communicated to the external advisors [27].

### Item 1c

Extent to which treatment was varied.

#### Explanation

The extent to which the treatment was individualised, both between patients and between practitioners, should be described. Trial protocols choose one of three broad levels of individualisation, ranging from none at all (all patients receiving the same treatment at all sessions), through partially individualised treatments (e.g. use of a fixed set of points to be combined with a set of points to be used flexibly), to fully individualised treatment protocols within which each patient receives a unique and evolving diagnosis and treatment. Additionally, the practitioners may have to apply a standardised theoretical framework, or may be allowed to apply their own. Many styles of acupuncture, whether based on traditional theories or Westernized concepts such as trigger points, are individualised in routine practice. Trials that are more pragmatic [28] in their aim, and designed to replicate routine settings and patient groups, have more of an emphasis on fully individualised treatment. In such cases standardisation may consist of a protocol that instructs practitioners to provide treatments as they normally do. Trials that are more explanatory (mechanistic) in their aim tend to need a tighter definition of specific components in order to minimise variation across treatments.

#### Examples

(i) Each patient received individualized acupuncture treatments that focused on specific needs and symptoms that the individual was experiencing. The rationale for this intervention was to test acupuncture as it is typically performed in practice. Point selection was based on the general principles of acupuncture and Traditional Chinese Medicine.(ref) The treatment was modified over the course of the study to accommodate the individual's changing pattern of pain, sleep, or other health issues [29].

(ii) The verum points consisted of obligatory points and additional points individually chosen by the physicians on the basis of traditional Chinese medicine diagnosis for syndromes (including tongue diagnosis), acupuncture channels related to the individual headache area, and Ah Shi points (locus dolendi points) [30].

(iii) The acupuncture protocol was based on the concept of adequacy of treatment,(ref) survey results,(ref) a consensus workshop, and recommendations from traditional Chinese protocols. We did not allow moxibustion, cupping, herbs, or electroacupuncture. For each individualised treatment session between six and 10 acupuncture points from 16 commonly used local and distal points were selected. Local points were Sp 9, Sp 10, St 34, St 35, St 36, Xiyan, Gb 34, and trigger points. Distal points were LI 4, TH5, Sp 6, Liv 3, St 44, Ki 3, BI 60, and Gb 41 [31].

## Stricta Item 2: Details of Needling

### Item 2a

Number of needle insertions per subject per session (mean and range where relevant).

#### Explanation

It is recommended that the reporting of this item should include a total of needle insertions per subject per session. This item is relevant to all designs of randomised controlled trials, from pragmatic to explanatory. For more explanatory designs where a formula of points is prescribed, the number of needle insertions should be reported as a simple total. For more pragmatic designs, with individualised treatments, the mean and range should be reported. Clearly, full details of individualised treatment cannot be reported in every section of Item 2 below. However, each item should be considered and as much information given as possible.

#### Examples

(i) The protocol allowed for up to 10 treatments per patient, the precise number being agreed between patient and practitioner. A total of 1269 treatments were provided, an average of 8.6 treatments per patient (range 1–10) and 9.6 needles per treatment (range 6–12). See (table) for variations between practitioners [32].

(ii) Disposable stainless steel needles (0.2×50mm, Seirin) were inserted into the skin over the trigger point to a depth of 10–30mm, appropriate to the muscle targeted, attempting to elicit a local muscle response using the “sparrow pecking” technique. After the local twitch response was elicited or a reasonable attempt made, the needle was retained for a further ten minutes. The mean number of insertions was 3.3 [33].

(iii) In the real acupuncture group, the acupuncture points *Hegu* (LI 4), *Jiache* (St 6), *Xiaguan* (St 7) and *Yifeng* (SJ 17) were used unilaterally on the tooth extraction side [34].

### Item 2b

Names (or location if no standard name) of points used (uni/bilateral).

#### Explanation

The point descriptions in the seminal classic texts, such as the Huangdi Neijing (Inner Canon of the Yellow Emperor) are rare and vague. The depiction of acupuncture points in relation to precise anatomical structures dates back only 100 years. Since the mid 1950s a process of standardisation has been taking place, and the acupuncture point descriptions based on anatomical locations and proportional *cun* measurement systems have served as a blueprint for many Western translations. It should be noted that these locations have not been universally adopted. Given this historical context, it remains important to know which acupuncture points have been used in clinical trials, with as accurate descriptions as possible of the location of these points, and where relevant the method used to identify the points.

The specific point locations used in treatments where standardised should be described in terms of an accepted nomenclature (e.g. GB21) [35] or in terms of anatomical location where there is no accepted name. Whether the needles are inserted unilaterally or bilaterally should be stated. For protocols with partially individualised prescriptions, list any prescribed essential or optional points, and describe (in the Results section) both the points used at every visit, and all the points used on an *ad hoc* basis. If the list is extensive, the most commonly used points (with percentages) should be reported. Where protocols specify using fully individualised point prescriptions, authors should consider the best way to report the points used, for example by listing all points across all subjects, or by identifying the most commonly used points if the list is extensive.

#### Examples

(i) We based the acupuncture point selections on Traditional Chinese Medicine meridian theory to treat knee joint pain, known as the “Bi” syndrome. These points consisted of 5 local points (Yanglinquan [gall bladder meridian point 34], Yinlinquan [spleen meridian point 9], Zhusanli [stomach meridian point 36], Dubi [stomach meridian point 35], and extra point Xiyan) and 4 distal points (Kunlun [urinary– bladder, meridian point 60], Xuanzhong [gall bladder meridian point 39], Sanyinjiao [spleen meridian point 6], and Taixi [kidney meridian point 3]) on meridians that traverse the area of pain (refs). The same points were treated for each affected leg. If both knees were affected, 9 needles were inserted in each leg [21].

(ii) The VA (verum acupuncture) group received acupuncture with a 0.25×40-mm stainless steel needle (Asia Med, Munich, Germany) at LI4, which is situated between the first two metacarpal bones on the dorsal side of both hands at the top of the muscle belly (figure provided) [36].

(iii) The most frequently treated local points were Bl 23, Bl 25, Gb 30, DU 4, Bl 26, and the extra point Huatuojiaji (table provided) ..….. The most frequently treated distant points were Bl 40, Kid 3, Gb 34, Bl 60, SI 3, and DU 20. In most cases, 8 to 12 local points and 4 to 6 distant points were used. Physicians used additional acupuncture points in 565 of the treatment sessions. The most frequently used additional local points were Li 4, St 40, Bl 17, Sp 6, and St 36 [27].

### Item 2c

Depth of insertion, based on a specified unit of measurement, or on a particular tissue level.

#### Explanation

The depth of insertion should be expressed using the Chinese measurement of the *cun*; in terms of anatomical depth, for example, of subcutaneous tissue, fascia, muscle or periosteum; or in millimetres. For some trials, the protocol might specify the angle and direction of insertion along with depth of insertion, in which case these should also be reported.

#### Examples

(i) All needle placements were performed by an experienced acupuncturist at a premarked depth of 4 mm from the tip of the needle [37].

(ii) The depth of needle insertion varied with thickness of the skin and subcutaneous fatty tissues at the site of the acupuncture points; it was usually 1 to 1.5cm [38].

(iii) Shallow and light needling stimulation (1–2 mm) using fine needles (0.18–0.16 mm) inserted with the aid of insertion tubes was emphasized. Points were needled at a 10°–20° angle with a 2-hand needling technique, generally in the direction of the flow of the channel [39].

### Item 2d

Responses sought (e.g., de qi or muscle twitch response).

#### Explanation

If the study protocol requires that specific responses to needling be elicited, for example the *de qi* sensation in traditional Chinese acupuncture, the muscle twitch in trigger point treatment or muscle contraction in electro-acupuncture, these elicited responses should be reported. Where relevant, the authors should differentiate between the responses required in the protocol and those actually obtained (which should be reported in the Results section).

#### Examples

(i) The TRP (trigger point) group received treatment at trigger points. The correct application of the technique requires experience in palpation and localisation of taut muscle bands and myofascial trigger points. Precise needling of myofascial trigger points provokes a brief contraction of the muscle fibres. This local twitch response must be elicited for successful therapy but it may be painful and post treatment soreness is frequent [33].

(ii) In contrast with TCM style acupuncture, we did not employ vigorous manipulation in order to elicit a strong *de qi* sensation (defined as a feeling of heaviness around the acupuncture point).(ref) Practitioners focused instead on feeling the response to stimulation as an “echo” sensation experienced on the receiving hand, while the active hand performed the actual needling. Attention was placed on reactivity or change in diagnostic areas, especially the pulse and abdomen. By carefully assessing changes in palpatory findings, the treatment was adjusted continuously based on the patient's response. Before needling, the “live” points were identified by palpation, that is, subtle changes at the skin level, or upon touch or pressure, for that particular patient [39].

### Item 2e

Needle stimulation (e.g. manual or electrical).

#### Explanation

Needle stimulation techniques, where used, should be clearly described for all points. For manual stimulation, such techniques include lifting, thrusting or rotating the needle to manipulate the de qi sensation. For electrical stimulation, the current, amplitude and frequency settings should be recorded.

#### Examples

(i) This mode of (manual) stimulation was provided via the acupuncture needles, which were placed in the premarked depth at the marked sites. The needle was rotated by an experienced acupuncturist with the index finger and thumb in an alternating clockwise and counterclockwise fashion at the rate of three to five rotations per second [37].

(ii) Electrical stimulation was given to the anterior part of the knee for 10 minutes and then 10 minutes for the posterior part using a battery-operated, four-channel, ‘AS Super 4’ Electrostimulator (RDG Medical, Surrey UK) which generated low frequency, square-wave (2–10Hz) pulses of 1 millisecond duration for 10 minutes.(ref) In both groups, the apparatus was attached to needles at the two *Xiyan* points, SP9 and GB34, and BL40 and BL57. Electrical stimulation was delivered at 6Hz at a constant current. Voltage was set at a level just above the pain threshold [38].

### Item 2f

Needle retention time.

#### Explanation

Needle retention times should be reported as either a standard or a mean and range. Authors should make it clear that they are reporting the time elapsed between the insertion and removal of needles (retention time) and distinguish it from treatment time, which may include other procedures such as history taking, discussion and preparation for treatment.

#### Examples

(i) Each participant was treated bilaterally and had a total of six needles inserted for the duration of the treatment. A draining technique was used and the needles were left for a period of 30 minutes. The practitioner returned to check on the participant at regular intervals during the intervention [40].

(ii) Needles were withdrawn immediately for tonification, and retained for up to 20 minutes for the evens technique [23].

(iii) Therapists allow 25 (min) to 35 (max) minutes between insertion of the last needle and cessation of treatment and during that time they are to revisit the needles as appropriate [41].

(iv) The patients in group A were dry needled for a few seconds. For trigger point inactivation by dry needling… it is especially important not to apply too strong a stimulus because this may produce a flare-up of the patient's symptoms [42].

### Item 2g

Needle type (diameter, length, and manufacturer or material).

#### Explanation

Details should be given of the types of needles used, including the diameter and length as well as the manufacturer and/or the material. This information is of importance since the effect of different metals or needle sizes on the body is not known. For trials using a variety of different types of needles, the ranges of diameters and lengths as well as types of material should be reported.

#### Examples

(i) Seirin 36 gauge 2.5 inches long unused sterile L-type needles were used for the study [37].

(ii) The VA (verum acupuncture) group received acupuncture with a 0.25×40-mm stainless steel needle (Asia Med, Munich, Germany) at LI4 [36].

## Stricta Item 3: Treatment Regimen

### Item 3a

Number of treatment sessions.

#### Explanation

The planned number of sessions and frequency of treatment should be clearly documented. The actual number of treatments received by participants should be reported in the Results section. If there is variation between patients, then the mean and range should be reported.

#### Examples

(i) The true acupuncture (experimental) group underwent 26 weeks of gradually tapering treatment according to the following schedule: 8 weeks of 2 treatments per week followed by 2 weeks of 1 treatment per week, 4 weeks of 1 treatment every other week, and 12 weeks of 1 treatment per month [21].

(ii) In all groups, participants were asked to attend treatment sessions twice weekly for 12 weeks (24 treatments). We considered participants who attended 80% or more (≥19 of 24) of acupuncture sessions to have completed a full course of treatment [43].

### Item 3b

Frequency and duration of treatment sessions.

#### Explanation

The frequency and duration of sessions should be documented, with mean and range to be reported where there is variation across patients. Any variation in frequency of treatment (for example if subjects are to be treated twice weekly in the first two weeks then once a week for the next six weeks) should be clearly reported.

#### Examples

(i) Acupuncture was administered a maximum of eight times, twice during each of the first three weeks and once during each of the following two weeks, for 30 minutes at each session. One month after this series of treatments had been completed and evaluated, the patients were offered a maximum of two follow up treatments of the same kind, one week apart [44].

## Stricta Item 4: Other Components of Treatment

### Item 4a

Details of other interventions administered to the acupuncture group (e.g. moxibustion, cupping, herbs, exercises, lifestyle advice).

#### Explanation

Additional components of treatment refer to the auxiliary techniques, prescribed self-treatment and lifestyle advice provided by the practitioner. All additional components, whether carried out by the practitioner or patient and whether integral or adjunctive to the acupuncture needling, should be described clearly. For acupuncture related interventions, such as moxibustion or cupping, detail should be provided equivalent to that recommended for acupuncture needling. If the protocol specifies the options of prescribed self-help treatments such as qigong or muscle stretching exercises, and/or lifestyle advice such as dietary changes based on acupuncture-related diagnostic criteria, then these too must be reported. The frequency with which the advice was given, and participants' compliance with this advice, should be reported. “Other components of treatment” should be distinguished from “co-interventions”, that is interventions that are provided additionally to both groups, which should be fully reported as described in STRICTA Item (6b) below.

#### Examples

(i) In addition to needling, moxibustion or thermal stimulation of the acupoints was used forming very fine wool of mugwort (*Artemisa vulgaris*) into minute, thread-size punks (*okyū*) and placing them on a thin layer of an herbal cream (*shiunko*). The moxa was lit with an incense stick and the process was repeated several times until warmth was felt by the patient [39].

(ii) Following application of the studs, patients were instructed to apply pressure to the stud by making small circular movements with the fingers of the opposite hand, 2–3 cycles per second for 1–2 minutes per point. As is typical for self-administered acupressure, patients were encouraged to apply acupressure this way on waking, in the early afternoon and during any exacerbation of symptoms. Initial instruction was provided verbally, at which time patients were asked to confirm their understanding by demonstrating the procedure. Patients also were given easy-to-read written materials describing the acupressure procedure [45].

(iii) Chinese herbal medicine was to be taken three times per day over a period of 6 weeks and parallel to acupuncture treatment… All herbs used in the present study were imported from China by a single TCM herbal medicine import company (Sinores, Lueneberg, Germany)… All herbs were prepared in dried, minced pieces and then sealed in generic paper sachets by a pharmacist in order to render the herbal formulation non-identifiable for patients… In addition to the basic formula, every patient received a second additional formula tailored to his or her individual TCM diagnosis [46].

### Item 4b

Setting and context of treatment, including instructions to practitioners and information and explanations to patients.

#### Explanation

The setting and context of treatment can also provide important additional components to treatment [47]. Context includes instructions to practitioners that might modify their normal practice, for example, prescribing or proscribing explanations to patients about their diagnosis. For patients, the context includes the information they have been given about the trial that might be expected to modify outcomes. Therefore, the information that the patient receives regarding the treatment and control intervention should be reported, including any relevant wording on consent forms and information leaflets designed to influence beliefs or expectations. For example, describing a sham acupuncture control as “a type of acupuncture” may have a different effect on outcome than saying it is “not acupuncture, but will involve a similar experience to acupuncture.”

#### Examples

(i) The first acupuncturist was the “diagnosing acupuncturist” (DA), whom the patient saw for the initial consultation, and before and after each treatment. A full case history was taken by the DA, together with tongue and pulse examination, to arrive at an individual diagnosis in accordance with the principles of TCM, with an additional lesser emphasis on Five Element Acupuncture (refs). Although all patients in the study had IBS, this corresponded to a wide range of TCM patterns, making individual diagnosis essential. Dietary and lifestyle advice (important in treatment according to TCM principles) was given to all patients by the DA, who then selected acupuncture points. The second “treating acupuncturist” (TA) opened the randomization envelope, and for the duration of the study remained the only individual aware of treatment allocation. The TA carried out the treatment - either according to instructions issued by the DA or using sham points, depending on the randomization [48].

(ii) Patients were informed about acupuncture and minimal acupuncture in the study as follows: “In this study, different types of acupuncture will be compared. One type is similar to the acupuncture treatment used in China. The other type does not follow these principles, but has also been associated with positive outcomes in clinical studies” [27].

## Stricta Item 5: Practitioner Background

### Item 5

Description of participating acupuncturists (qualification or professional affiliation, years in acupuncture practice, other relevant experience).

#### Explanation

Characteristics of the acupuncturists providing treatment should be reported, including qualification or affiliation, years in acupuncture practice, as well as any other experience that may be relevant to the trial. Relevant differences (if any) in the qualification, training and experience of the participating acupuncturists should be highlighted. The recent survey of authors of acupuncture trials and reviews reinforced the need for these characteristics to be reported well [12], especially since the actual level of reporting has historically been poor [13]. In trials where different acupuncturists provide treatment to different treatment arms, the background of both groups should be reported. The eligibility criteria for acupuncturists should be explained, as these will influence generalisability of the trial results. Where there are known to be potential variations between practitioners, selecting a random sample of practitioners will reduce expertise bias and help improve the applicability of the results [49].

#### Examples

(i) Physicians had a median of 350 hours (range 140–2508 hours) of acupuncture training before participating in the trial; 33 (73%) had the B-Diploma. Seventeen (17; 38%) trial physicians taught acupuncture in accredited postgraduate courses. The physicians had used acupuncture in their practices for an average of 11 years (median 10, range 0–25) and had treated 346 patients (range 22–1200) with acupuncture in the year before the trial. Forty-one physicians (92%) indicated that they frequently or always make a Chinese syndrome diagnosis before starting treatment [27].

(ii) Eight US-trained and licensed acupuncturists with a median of 10 years of experience (range 4–18 years) provided study treatments in their private offices. One investigator trained the acupuncturists in the study procedures to increase their comfort with delivering all 4 treatments and monitored compliance with the protocol throughout the study [43].

(iii) Of the 11 midwives participating in the study, six had been taught acupuncture for midwives at the Norwegian School of Acupuncture/NFKA. These six gave real and false acupuncture, whereas the others, who had been trained in acupuncture by the six, were allowed only to give false acupuncture [50].

## Stricta Item 6: Control and Comparator Interventions

### Item 6a

Rationale for the control or comparator in the context of the research question, with sources that justify the choice(s).

#### Explanation

The rationale for choice of control or comparator should be presented and justified in relation to the research question and the methodology. In studies in which a group receiving acupuncture is compared with another group, the control or comparator can be sham acupuncture, usual care, an active treatment, a waiting list or no treatment. Whereas ‘control’ is sometimes used for a group that receives no intervention, the term ‘comparator’ may be more appropriate for an active intervention, such as physiotherapy, for which the intended action of the comparator is expected to be therapeutic. If using an acupuncture-like control in a participant-blinded trial then one of the following terms: active acupuncture control; penetrating needle control; or non-penetrating sham needling control might be helpful descriptors. Control procedures involving invasive or non-invasive sham needling techniques may be therapeutically active, evoking neurophysiological and/or localised immune and circulatory responses. The extent that sham acupuncture needling, whether penetrating or not, might elicit acupuncture-specific physiological mechanisms is not known, and is in part a consequence of our lack of knowledge of the mechanism(s) of true acupuncture. There are also variations in assumptions about the precision required for point location, as for some clinicians and investigators, acupuncture points are considered as areas of reactivity rather than points of action. Such assumptions have a bearing on the integrity of the sham as an appropriate control. Some non-needling control procedures can be assumed to be physiologically inert, such as an inactivated transcutaneous electrical nerve stimulation (TENS) machine; however, these procedures may not have the same total psychophysiological credibility as acupuncture, thereby compromising the interpretation of the results. Sources that led to the choice of control, such as literature or expert opinion, should also be reported and referenced. The author should reference prior work that supports the use of the selected comparator, such as from the conclusion of a systematic review or from another randomised controlled trial.

#### Examples

(i) ‘Sham’ acupuncture points were chosen from three different areas on the body (the anterior thigh distally, the posterior thigh, and the lateral aspect of the lower back), which do not correspond to recognized acupuncture points and are deemed to have no therapeutic value [48].

(ii) International guidelines suggest that the best package of care for this patient group is one that includes patient education, advice and exercise (ref). …. Randomised clinical trials consistently show the benefit of exercise for knee pain in older adults (refs). Recent studies also highlight the need to provide adequate instruction, feedback and practice in order to ensure that the key muscle groups around the knee, such as the quadriceps, are activated (ref). The European League Against Rheumatism (EULAR) recommendations have recently been updated and in particular, advocate exercise for knee pain related to osteoarthritis (ref). In line with this evidence base, the current trial was designed so that all participants receive a package of care which includes education, advice, and exercise [41].

(iii) For this study a special ‘placebo needle’ was designed by Streitberger. The needle body is not fixed inside the copper handle. Its tip is blunt and when it touches the skin, a small pricking sensation is felt by the patient, simulating the puncture of the skin. The handle of the needle moves over the needle, the needle is shortened. Patients ‘see’ the needle moving inside their body… This needle was tested in 60 volunteers and proved to be sufficiently credible to be used in our clinical trial as a control(ref)[51].

### Item 6b

Precise description of the control or comparator. If sham acupuncture or any other type of acupuncture-like control is used, provide details as for Items 1 to 3 above.

#### Explanation

A precise description of the components of the control or comparator should be presented. If the control treatment is an acupuncture-like intervention, such as a form of sham acupuncture, then it should be specified whether the sham is invasive (penetrating the skin) or non-invasive (non-penetrating). The theoretical basis, needling details and regimen of an acupuncture-like control need to be reported in the same way as is set out in STRICTA Items 1 to 3 above. The lack of a world-wide consensus on the location and size of acupuncture points reinforces the importance of accurate documentation of the sham points actually used, their precise location and the method used to locate them. If usual care or another active treatment is the comparator, all the components should be reported in full detail. This will enable readers to compare usual care as provided in the trial with what is usually provided to participants in another setting. Where usual care is also provided to those receiving acupuncture, these data will also allow readers to compare the intensity of usual care in the comparator arm with that of the experimental arm. If it is a waitlist arm, then the period of waiting needs to be specified. While precise description of the control or comparator is fairly straightforward in principle, the more complex the components, the more care is required to specify them precisely.

#### Examples

(i) Acupuncturists inserted 2 needles into the sham points in the abdominal area, approximately 3 cm lateral to and slightly above the umbilicus bilaterally, and then immediately applied 2 pieces of adhesive tape next to the needles. In addition, they tapped a mock plastic needle guiding tube on the surface of each of the 9 true points in the leg to produce some discernible sensation and then immediately applied a needle with a piece of adhesive tape to the dermal surface, without needle insertion, of each point for a total of 20 minutes. The sham acupuncture procedure was given on the same schedule as the experimental group and used the same active needle placements, except actual insertion did not occur at these 9 points. Although electrical stimulation did not occur, a mock transelectrical stimulation unit (which emitted a sound and possessed a blinking light) was attached to the sham needles at the knee. To facilitate blinding, we used screens in both treatment and sham groups that were placed below the abdomen to prevent participants from actually observing the true or sham procedures at the knee area but to allow them to observe the procedure being performed in the abdomen area [21].

(ii) In each session, at least 5 out of 10 predefined distant nonacupuncture points (ref) were needled bilaterally (at least 10 needles) and superficially using fine needles (ie, minimal acupuncture). “De Qi” and manual stimulation of the needles were avoided. All acupuncturists received oral instructions, a videotape, and a brochure with detailed information on sham acupuncture [52].

(iii) Conservative therapy involved 10 visits to practitioners with consultation and a prescription for diclofenac, up to 150 mg/d, or rofecoxib, 25 mg/d, as needed until week 23 [53].

(iv) Patients received the same treatment as in the standard group but in addition did stabilising exercises modified because of the pregnancy.(refs) The training programme started by emphasising activation and control of local deep lumbopelvic muscles. Training of more superficial muscles in dynamic exercises to improve mobility, strength, and endurance capacity was gradually included. Patients received treatments individually for a total of six hours during six weeks. They were told to integrate the exercises in daily activities and to exercise in short sessions on several occasions during the day [54].

## Discussion

This revised STRICTA Statement has been designed to help improve the reporting of interventions in clinical trials of acupuncture, with the intention that it will help authors of acupuncture trials provide readers with a clear, accurate and transparent account of their acupuncture protocols as well as their control and/or comparator procedures. In addition to revising the STRICTA checklist, we have improved the explanations of each item and provided examples of good reporting. To enhance awareness, endorsement and adherence, the revised STRICTA Statement has been developed as an extension to CONSORT. Authors of clinical trials of acupuncture should use the STRICTA recommendations for the acupuncture intervention (Item 5 in the CONSORT 2010 Statement) in conjunction with the other 25 items of the checklist in the main CONSORT guidelines [10],[11]. The extension to CONSORT for non-pharmacological interventions is also highly relevant to acupuncture trials [14],[15]. There are other extensions to CONSORT that may be relevant, depending on the type of trial design, including extensions for cluster trials, equivalence and non-inferiority trials, and pragmatic trials, and the reporting of abstracts and of harms (e.g. adverse events) associated with the intervention. The most recent versions of all CONSORT guidance documents can be found on the CONSORT website (http://www.consort-statement.org).

A complete, accurate and transparent trial report facilitates dissemination, interpretation, translation and replicability. There continues to be a need for better reporting generally, as has been highlighted in a recent study of what is missing from descriptions of treatments in trials and reviews [55]. The authors found that elements of the intervention were missing in half of the published articles that they reviewed, giving insufficient detail, for example, with practitioners unable to use the treatments as described and researchers unable to replicate studies. This finding is similar to that from a review of authors of acupuncture trials [12]. Improved reporting reduces reader ambiguity in interpretation, is likely to increase credibility and application of the results by providing better evidence on which to base decisions on patient care.

Reporting guidelines do help improve the quality of reporting randomized trials [56] although it is difficult to observe their maximal benefit because too few journals endorse reporting guidelines [57] and fewer adhere to them [57]. To maximize this potential, we encourage journals to unambiguously endorse the revised STRICTA reporting guidelines. This can be most readily achieved by updating journal Instructions to Authors, thereby alerting prospective authors. In addition, we encourage journals to implement strategies to improve author adherence to reporting guidelines. These efforts might also help peer reviewers and journal editors in deliberating the merits of such trials.
